# Study protocol: differential effects of diet and physical activity based interventions in pregnancy on maternal and fetal outcomes—individual patient data (IPD) meta-analysis and health economic evaluation

**DOI:** 10.1186/2046-4053-3-131

**Published:** 2014-11-04

**Authors:** Anneloes E Ruifrok, Ewelina Rogozinska, Mireille NM van Poppel, Girish Rayanagoudar, Sally Kerry, Christianne JM de Groot, SeonAe Yeo, Emma Molyneaux, Fionnuala M McAuliffe, Lucilla Poston, Tracy Roberts, Richard D Riley, Arri Coomarasamy, Khalid Khan, Ben Willem Mol, Shakila Thangaratinam

**Affiliations:** 1 Department of Obstetrics and Gynecology, Academic Medical Centre, Amsterdam, The Netherlands; 2 Department of Obstetrics and Gynaecology, Faculty of Medicine, VU University Medical Center, Amsterdam, The Netherlands; 3 Women’s Health Research Unit, Barts and The London School of Medicine and Dentistry, Queen Mary University of London, London, UK; 4 Department of Public and Occupational Health, EMGO + Institute for Health and Care Research, VU University Medical Center, Amsterdam, The Netherlands; 5 Multidisciplinary Evidence Synthesis Hub (mEsh), Barts and The London School of Medicine and Dentistry, Queen Mary University of London, London, UK; 6 School of Nursing, University of North Carolina at Chapel Hill, Chapel Hill, NC, USA; 7 Section of Women’s Mental Health, Health Service and Population Research Department, Institute of Psychiatry, King’s College London, London, UK; 8 School of Medicine & Medical Science, UCD Institute of Food and Health, Dublin, Ireland; 9 Division of Women’s Health, Women’s Health Academic Centre, King’s College London, St. Thomas’ Hospital, London, UK; 10 Health Economics Unit, School of Health and Population Sciences, College of Medical and Dental Sciences, University of Birmingham, Birmingham, UK; 11 School of Health and Population Sciences, College of Medical and Dental Sciences, University of Birmingham, Edgbaston, Birmingham B15 2TT, UK; 12 School of Clinical and Experimental Medicine, College of Medical and Dental Sciences, University of Birmingham, Birmingham, UK; 13 School of Paediatrics and Reproductive Health, Robinson Institute, University of Adelaide, Adelaide, Australia

**Keywords:** Individual patient data meta-analysis, Diet and physical activity, Pregnancy, Weight gain

## Abstract

**Background:**

Pregnant women who gain excess weight are at risk of complications during pregnancy and in the long term. Interventions based on diet and physical activity minimise gestational weight gain with varied effect on clinical outcomes. The effect of interventions on varied groups of women based on body mass index, age, ethnicity, socioeconomic status, parity, and underlying medical conditions is not clear. Our individual patient data (IPD) meta-analysis of randomised trials will assess the differential effect of diet- and physical activity-based interventions on maternal weight gain and pregnancy outcomes in clinically relevant subgroups of women.

**Methods/design:**

Randomised trials on diet and physical activity in pregnancy will be identified by searching the following databases: MEDLINE, EMBASE, BIOSIS, LILACS, Pascal, Science Citation Index, Cochrane Database of Systematic Reviews, Cochrane Central Register of Controlled Trials, Database of Abstracts of Reviews of Effects, and Health Technology Assessment Database. Primary researchers of the identified trials are invited to join the International Weight Management in Pregnancy Collaborative Network and share their individual patient data. We will reanalyse each study separately and confirm the findings with the original authors. Then, for each intervention type and outcome, we will perform as appropriate either a one-step or a two-step IPD meta-analysis to obtain summary estimates of effects and 95% confidence intervals, for all women combined and for each subgroup of interest. The primary outcomes are gestational weight gain and composite adverse maternal and fetal outcomes. The difference in effects between subgroups will be estimated and between-study heterogeneity suitably quantified and explored. The potential for publication bias and availability bias in the IPD obtained will be investigated. We will conduct a model-based economic evaluation to assess the cost effectiveness of the interventions to manage weight gain in pregnancy and undertake a value of information analysis to inform future research.

**Systematic review registration:**

PROSPERO 2013: CRD42013003804

## Background

Excessive weight gain in pregnancy is associated with maternal and fetal complications such as pre-eclampsia, gestational diabetes, caesarean section, large for gestational age babies [1-8], and postpartum weight retention [9,10]. It is a risk factor for maternal and childhood obesity in the long term [5,9,11], resulting in significant burden to the health care systems globally [9,10,12-17]. In the UK, obesity costs the National Health Service (NHS) around £4 billion a year and the economy a further £16 billion in indirect costs [18,19]. Reducing excessive weight gain in pregnancy by effective weight management programmes could lead to significant societal advantages in terms of health and costs.

In the antenatal period, women are in regular contact with health professionals and are highly motivated to make changes that may improve their pregnancy outcomes [20]. Our study-level meta-analysis of 44 randomised trials showed that dietary and lifestyle interventions were effective in reducing weight gain in pregnancy and reduced risk of adverse outcomes [21]. We were restricted by unexplained heterogeneity of effects and paucity of published detail from making firm recommendations for clinical practice, especially for pregnancy outcomes. Importantly, we were unable to ascertain if the intervention had a differential beneficial effect on particular subgroups of women.

The only guidance on weight gain recommendations in pregnancy is by the Institute of Medicine (IOM) in the US, which is based on observational evidence. The UK and other European policy makers do not recommend specific weight gain targets in pregnancy due to the absence of robust evidence. The Public Health Interventions Advisory Committee (PHIAC) in the UK has prioritised the need for research to identify the most effective and cost-effective ways of helping women to manage their weight during pregnancy, including women who are obese, those who are under 18 and those from disadvantaged, low income and minority ethnic groups [22]. Additionally, they highlighted the need to ascertain whether adherence to IOM recommendations on gestational weight gain improves obstetric outcomes, especially in minority groups and teenagers.

We plan to undertake an individual patient data (IPD) meta-analysis [23] to robustly address the above questions on the effect of weight management interventions in women stratified by BMI, ethnicity, socioeconomic status and teenage pregnancies by obtaining raw patient-level data for synthesis across trials.

### Objectives

The primary objective of this IPD meta-analysis is to determine the differential effects of weight management interventions in pregnancy on maternal weight gain and composite adverse maternal and fetal outcomes in women according to their (i) body mass index (BMI), (ii) age, (iii) ethnicity, (iv) parity, and (v) underlying medical conditions.

The secondary objectives are to:

i. Validate weight change as an outcome measure by quantifying the relationship between the amount of weight gained in pregnancy and the risk of adverse maternal and fetal outcomes for normal weight (BMI 18.5–24.9 kg/m^2^), overweight (BMI 25–29.9 kg/m^2^) and obese (BMI ≥30 kg/m^2^) women

ii. Assess if adherence in pregnancy to IOM weight gain recommendations minimises adverse pregnancy outcomes in normal weight, overweight and obese women

iii. Identify the prognostic factors for gestational weight gain based on patient characteristics such as pre-pregnancy BMI, age, ethnicity, socioeconomic status, parity, ethnicity, smoking, diet and lifestyle

iv. Undertake network meta-analysis to produce a rank order of interventions

v. Assess the cost effectiveness of the various interventions in pregnancy using model-based full economic evaluation with value of information (VOI) analysis

## Methods/design

Our IPD meta-analytical approach will follow existing guidelines, and our output will comply with the PRISMA statement and adhere to recent reporting guidelines for IPD meta-analysis.

### Search strategy

We will update the literature search to identify new trials published since the completion of our systematic review (HTA No. 09/27/06) on effects of weight management interventions in pregnancy [21]. The following databases will be searched: MEDLINE, EMBASE, BIOSIS, LILACS, Pascal, Science Citation Index, Cochrane Database of Systematic Reviews (CDSR), Cochrane Central Register of Controlled Trials (CENTRAL), Database of Abstracts of Reviews of Effects (DARE) and Health Technology Assessment Database (HTA). Other relevant databases including the Inside Conferences, Systems for Information in Grey Literature (SIGLE), Dissertation Abstracts, and Clinical Trials.gov will be searched. Internet searches will include specialist search gateways (such as OMNI: http://omni.ac.uk), general search engines (such as Google: https://www.google.co.uk), and meta-search engines (such as Copernic: http://www.copernic.com). In addition, information on studies in progress, unpublished research, research reported in the grey literature, and details from commercial providers will be sought. Language restrictions will not be applied. The search will be updated 1 year before the end of the project to avoid missing recently published studies.

### Establishment of the International Weight Management in Pregnancy IPD Collaboration

We contacted researchers who have published trials on weight management interventions in pregnancy and established the International Weight Management in Pregnancy (i-WIP) IPD Collaborative Network [21]. There has been an overwhelming interest for a joint endeavour in this field. The network, supported by WHO (World Health Organization), is a global effort in bringing together researchers, clinicians and epidemiologists from 14 countries ( https://kamolo.org.ar/iwipipd) Thirty-six collaborators have joined the network to date providing access to anonymised individual data of 9,344 women (Table 1).

**Table 1:** Studies with provisional support and consideration to share individual patient data

**Study year**	**Country**	**Study characteristics**	**Outcomes**		**Sample size**
			**Maternal**	**Fetal**	
Althuizen 2012	Netherlands	Ethnically diverse; no BMI restrictions; age, nr; GA at inclusion, <14 weeks; glucose status, nr; other risk factors, nr	GWG, GDM, preterm delivery, CS	Birth weight, macrosomia	269
Barakat 2009	Spain	Caucasian; BMI restrictions, nr; age, 25–35 years; GA at inclusion, nr (total at least 26 weeks intervention); glucose status, nr; no known pre-existing health problems	GWG, GA, preterm delivery	Birth weight, LGA, SGA, AS, macrosomia (>4,000 g)	142
Barakat 2011	Spain	Spanish (White); BMI restrictions, nr; age, 23–38 years; GA at inclusion, first prenatal visit; glucose status, nr; no known pre-existing health problems	GWG, GA CS, vaginal delivery	Birth weight, AS	80
Barakat 2013	Spain	Caucasian; no BMI restrictions; age, nr; GA at inclusion, <10 weeks; glucose status, nr; no known pre-existing health problems	GWG, GA, GDM, PIH, preterm delivery	Birth weight, AS	765
Bogaerts 2012	Belgium	Ethnically diverse; BMI, ≥29 kg/m^2^; age, nr; GA at inclusion, <15 weeks; nondiabetic; other risk factors, nr	GWG, GA, PE, PIH, GDM, IOL, CS, vaginal delivery	Birth weight, AS	197
Cavalcante 2009	Brazil	Race, nr; no morbid obesity; age restrictions, nr; GA at inclusion, 16–20 weeks; glucose status, nr; no known pre-existing health problems	GWG, preterm delivery	Birth weight	71
Clapp 1997	USA	Race, nr; no morbid obesity; age restrictions, nr; GA at inclusion, 8 weeks; glucose status, nr; no known pre-existing health problems	GWG	Birth weight	51
Clapp 2000	USA	Race, nr; no morbid obesity; age restrictions, nr; GA at inclusion, 8 weeks; glucose status, nr; no known pre-existing health problems	GWG, GA	Birth weight	12
Dodd 2014	Australia	Race, nr; BMI, ≥25 kg/m^2^; age restrictions, nr; GA at inclusion, <20 weeks; nondiabetic; other risk factors, nr	PE, PIH, GDM, IOL, CS, preterm delivery	LGA, macrosomia (>4,000 g), hypoglycaemia, shoulder dystocia, admission to NICU	1,582
El Beltagy 2013	Egypt	Race, nr; BMI, obese; age restrictions, nr; GA at inclusion, first antenatal visit; glucose status, nr; other risk factors, nr	GWG, GDM	Birth weight, macrosomia	100
Grant 2013	Canada	Race, predominantly non-Caucasian; BMI restrictions, nr; age, >18 years, GA at inclusion, nr; glucose status, impaired glucose tolerance or GDM; no known pre-existing health problems	GWG	Birth weight, macrosomia	47
Guelinckx 2010	Belgium	Caucasian; BMI, ≥29 kg/m^2^; age restrictions, nr; GA at inclusion, <15 weeks; nondiabetic; no known pre-existing health problems	GWG, GA, PE, PIH, IOL, CS	Birth weight, LGA	85
Haakstad 2011	Norway	Race, nr; BMI restrictions, nr; age restrictions, nr; GA at inclusion, <24 weeks; glucose status, nr; no known pre-existing health problems	GWG		105
Hui 2006	Canada	Ethnically diverse; BMI restrictions, nr; age restrictions, nr; GA at inclusion, <26 weeks; nondiabetic; no known pre-existing health problems	GWG, GA, GDM	Birth weight, LGA	45
Hui 2011	Canada	Race, nr; BMI restrictions, nr; age restrictions, nr; GA at inclusion, 20–26 weeks; nondiabetic; no known pre-existing health problems	GWG, GA, GDM, CS	Birth weight, LGA	224
Jackson 2010	USA	Ethnically diverse; BMI restrictions, nr; age, >18 years; GA at inclusion, <26 weeks; glucose status, nr; other risk factors, nr	GWG		321
Jeffries 2009	Australia	Race, nr; BMI restrictions, none; age, >18 to <45 years, GA at inclusion, <14 weeks; nondiabetic; other risk factors, nr	GWG, PE, PIH, GDM , preterm delivery, CS	Birth weight, LGA, SGA, hypoglycaemia, shoulder dystocia	236
Khaledan 2010	Iran	Race, nr; BMI restrictions, nr; age restrictions, nr; GA at inclusion, 24–32 weeks; no diabetes mellitus type 1 (DM1) with poor control; no known pre-existing health problems	GWG, GA, CS	Birth weight	39
Khoury 2005	Norway	Caucasian; BMI, 19–32 kg/m^2^; age, 21–38 years; GA at inclusion, 17–20 weeks; nondiabetic; no known pre-existing health problems	GWG, PE, preterm delivery	Birth weight, SGA, intra-uterine death	290
Luoto 2011	Finland	Race, nr; BMI, >17 kg/m^2^; age, >18 years; GA at inclusion, 8–12 weeks; nondiabetic; no known pre-existing health problems	GWG, GA, PE, GDM	Birth weight, LGA, SGA	399
Nascimento 2011	Brazil	Race, nr; BMI, >26 kg/m^2^; age >18 years; GA at inclusion, 14–24 weeks; nondiabetic; no known pre-existing health problems	GWG, PIH, GDM, CS	Birth weight, AS, LGA, SGA	82
Ong 2009	Australia	Race, nr; obese; age restrictions, nr; GA at inclusion, 18 weeks; nondiabetic; other risk factors, nr	GWG		12
Oostdam 2012	Netherlands	Ethnically diverse; BMI, ≥25.0 kg/m^2^; age, >18 years; GA at inclusion, <20 weeks; nondiabetic; no known pre-existing health problems	GWG, GDM	Birth weight	124
Phelan 2011	USA	Ethnically diverse; BMI, ≥19.8–26.0 kg/m^2^; age >18 years; GA at inclusion, 10–16 weeks; glucose status, nr; no known pre-existing health problems	GWG, GA, PE, PIH, GDM, preterm delivery, CS	Birth weight, macrosomia, birth weight <2,500 g	401
Poston 2013	UK	Race, nr; BMI, ≥30 kg/m^2^; age restrictions, nr; GA at inclusion, >15^+0^ weeks and <17^+6^; nondiabetic; no known pre-existing health problems	GA, GWG, PE, GDM, mode of delivery	Birth weight, macrosomia, still birth	183
Prevedel 2003	Brazil	Race, nr; BMI restrictions, nr; age restrictions, nr (primiparous or adolescents); GA at inclusion, 16–20 weeks; glucose status, nr; no known pre-existing health problems	GWG, preterm delivery	Birth weight, SGA	132
Rauh 2013	Germany	Race, nr; BMI, ≥18.5 kg/m^2^; age, ≥18 years; GA at inclusion, <18 weeks; nondiabetic; no known pre-existing health problems	GWG, GDM, IOL, CS, preterm delivery	LGA, SGA	250
Sagedal 2014	Norway	Race, nr; BMI, ≥19 kg/m^2^; age, ≥18 years; GA at inclusion, <20 weeks; nondiabetic; no known pre-existing health problems	GWG, GDM, CS	LGA	600
Stafne 2012	Norway	White; no BMI restrictions; age, >18 years; GA at inclusion, 18–22 weeks; nondiabetic; no known pre-existing health problems	GA, PE, PIH, GDM, CS	Birth weight, AS, LGA, admission to NICU	124
Vesco 2013	USA	Race, nr; BMI, ≥30 kg/m^2^; age, nr; GA at inclusion, <20 weeks; nondiabetic; no known pre-existing health problems	GWG, GA, PE, PIH, GDM, CS, preterm delivery	Birth weight, LGA, SGA, macrosomia (4,000 g)	114
Vinter 2011	Denmark	Caucasian; BMI, 30–45 kg/m^2^; age, 18–40 years; GA at inclusion, 10–14 weeks; nondiabetic; no known pre-existing health problems	GWG, PE, PIH, GDM, CS	LGA, admission to NICU	855
Vitolo 2011	Brazil	Race, nr; BMI restrictions, none; age, <35 years; GA at inclusion, 10–29 weeks; nondiabetic; no known pre-existing health problems	GWG, PE, PIH, GDM, preterm birth	Birth weight	315
Walsch 2012	Ireland	Race, nr; BMI restrictions, nr; age, >18 years; GA at inclusion, <18 weeks; nondiabetic; no known pre-existing health problems	GWG, GA, preterm delivery, IOL, CS	Birth weight, macrosomia	304
Wolff 2008	Denmark	Caucasian; BMI, ≥30 kg/m^2^; age, >18 to <45 years; GA at inclusion, <18 weeks; nondiabetic; no known pre-existing health problems	GWG PE, PIH, GDM , CS	Birth weight	800
Yeo 2012	USA	Ethnically diverse; BMI, >19.8 kg/m^2^; no age restrictions; GA at inclusion, 18 weeks; nondiabetic; no known pre-existing health problems	GWG, PE, PIH	Birth weight	17

*AS* Apgar score, *CS* caesarean section, *GA* gestational age, *GDM* gestational diabetes mellitus, *GWG* gestational weight gain, *IOL* induction of labour, *LGA* large for gestational age, *NICU* Neonatal Intensive Care Unit, *nr* not reported, *PE* pre-eclampsia, *PIH* pregnancy-induced hypertension, *RDS* respiratory distress syndrome, *SGA* small for gestational age.

### Inclusion and exclusion criteria

Randomised controlled trials evaluating diet- and physical activity-based interventions in pregnancy compared to normal antenatal care are eligible for inclusion. Underweight women (BMI <18.5 kg/m^2^) and women with contra-indications to limit gestational weight gain will be excluded. The interventions include those that are based on diet, physical activity, or a mixed approach comprising diet and physical activity with or without behavioural modification techniques. Studies assessing weight-reducing drugs or surgical interventions will not be included.

### Outcome measures

The primary outcome measures are (i) maternal weight gain in pregnancy and (ii) composite adverse maternal and fetal outcomes. Gestational weight gain is defined as the change in weight from the first scheduled visit to the weight measured before delivery. The composite outcome includes those components that are critically important to clinical practice and whose underlying biology is similar [24]. The individual components were defined according to NICE guidelines [25,26] and identified by a four-round Delphi survey. The first two rounds of the survey identified the clinically important outcomes with input from experts [27]. The subsequent two rounds of the Delphi survey were completed by i-WIP collaborators to ensure that the outcomes were clinically relevant, of equal importance, occur with similar frequency and have the same direction of effect with the intervention (Table 2). We will include in our analysis those components for which robust data is available across the individual studies.

**Table 2:** Critically important maternal and fetal outcomes in the IPD meta-analysis of weight management interventions identified by the Delphi survey

**Maternal outcomes**	**Fetal outcomes**
Pre-eclampsia	Intrauterine death
Pregnancy induced hypertension	Small for gestational age
Gestational diabetes mellitus	Large for gestational age
Preterm delivery	Admission to NICU
Caesarean section	Shoulder dystocia
Thromboembolism	>1 perinatal complication
Admission to high dependency unit/intensive treatment unit	Birth trauma
Weight gain in pregnancy	Long-term neurological sequelae
	Long-term metabolic sequelae

### Study quality assessment and data collection

A bespoke database will be set up and authors will be allowed to supply data in whatever way convenient to them. We will consider all recorded variables, even those not reported in the published studies. The quality of each trial will be assessed [28,29] to evaluate the integrity of the randomisation and follow-up procedure. We will evaluate the risk of bias in individual studies by considering six items used in the Cochrane risk of bias tool: sequence generation, allocation concealment, blinding, incomplete outcome data, selective outcome reporting, and other potential sources of bias.

### Sample size considerations

Although no formal sample size requirements are necessary for the meta-analysis, we have considered the potential power of our IPD meta-analysis in comparison to single trials in this field to detect clinically important effects in each subgroup separately (Table 3). All calculations relate to a type I error of 5%, a power of 80% and a loss to follow-up of 5%. We chose a reduction of 2.5 kg in gestational weight gain as the minimally important difference (MID), since it was associated with improvement in obstetric outcomes [12]. Our sample size will be over 9,000 women. For maternal weight gain, the sample size required for all subgroups is 300 or less. Given the large sample size available, it is highly likely that the study is powered to detect important differences between subgroups (that is, to identify genuine factors that modify treatment effect). This will allow us to detect interaction terms as small as about 30% of the size of the overall treatment effect. So, if the overall intervention effect is a reduction in weight gain of 2.5 kg, then our IPD meta-analysis will have 80% power to detect an interaction term of about 2.5 × 0.3 = 0.75 or above (e.g. a difference in intervention effect of 0.75 kg between obese and normal weight women).

**Table 3:** Comparison of existing evidence on effectiveness of weight management interventions and the proposed IPD meta-analysis

**Characteristics**	**Existing systematic reviews**	**Published and ongoing primary studies**	**Proposed IPD meta-analysis**
Consistent inclusion and exclusion criteria e.g. BMI, risk status	x	√	√
Assessment of effect of prognostic factors on treatment effect e.g. diabetic status, chronic hypertension	x	√	√
Missing data observed and accounted at individual level	x	√	√
All critically important maternal and fetal outcomes considered	√	x	√
Potential for sufficient power to assess for differential treatment effect across groups e.g. BMI, ethnicity, race, parity	x	x	√
Standardisation of statistical analysis across studies	x	N/A	√
Correlation between multiple end points accounted e.g. each participant providing data on gestational weight gain in various trimesters and weight retention postpartum	x	√	√
Up to date follow-up information, potentially longer than that used in the original study publication	x	x	√

For the composite outcome of adverse maternal and fetal outcomes, we calculated the sample size needed to detect an intervention effect of a 30% reduction in adverse pregnancy outcome. Our estimates of the standard deviation (SD) of the control group and the risk of composite pregnancy outcome were obtained from the data of primary studies included in our systematic review. The largest sample size required is 2,330 for the adverse pregnancy outcome in the normal BMI group. Of our 5,000 women, we expect over half to be in this normal category. For overweight and obese women, the sample size required for the adverse pregnancy outcome is 1,290 and 770 respectively (Table 4).

**Table 4:** Sample size estimations evaluating the effect of weight management interventions

**Subgroups**		**Control group SD**	**Sample size required to detect a 2.5-kg reduction in gestational weight gain**	**Control group: probability of adverse maternal and fetal outcomes**	**Sample size required to detect a 30% reduction in adverse maternal and fetal outcomes**
BMI	Obese	7.5	300	0.30	770
	Overweight	7.5	300	0.20	1,290
	Normal	5.1	140	0.12	2,330
Age	<20 years	7.12	270		
	≥20 years	5.87	184		
Ethnicity	Caucasian	3.4	64		
	Asian	3.8	78		
	African	5.1	140		
Parity	<1	6.28	212		
	≥1	6.68	238		
Risk factors like diabetes	High risk	6.81	248		
	Low risk	6.67	236		

Table 1 shows a comparison of existing evidence on effectiveness of weight management interventions in pregnancy and the proposed IPD meta-analysis.

### Data analysis

#### Summarising the overall effect of weight management interventions

First, we will summarise the overall effect of each intervention (in relation to each outcome) across the entire set of available patient data. Meta-analyses of the effectiveness of weight management interventions in pregnancy will be performed for the weight-related and composite adverse maternal and fetal outcomes. We will include all patients ever randomised and will base our analysis on the intention to treat principle. Women with glucose intolerance will be excluded in the analysis of composite adverse pregnancy outcomes, as gestational diabetes is one of the components of the composite outcome.

All studies will be reanalysed separately and the original authors asked to confirm the individual study results, and any discrepancies will be resolved. Then, for each intervention type and outcome separately, we will perform either a one-step or a two-step IPD meta-analysis to obtain the pooled intervention effect. The one-step approach analyses the IPD from all studies simultaneously, whilst accounting for the clustering of patients within studies. In contrast, the two-step approach first estimates the intervention effect from the IPD in each study separately and then pools them using a conventional meta-analysis of the intervention effect estimates obtained. One-step and two-step meta-analyses usually give similar results but, where possible, will undertake both to ensure that conclusions remain robust to the choice of method [23,30].

Given the heterogeneity identified in our previous review [21], we also expect to observe significant heterogeneity in the IPD meta-analysis. Thus, we will use a random effects meta-analysis approach, which allows for between-study heterogeneity in intervention effect. Heterogeneity will be summarised using the *I*-squared statistic (which provides the proportion of total variability that is due to between-study heterogeneity) and the estimated between-study variance (‘tau-squared’), obtained using restricted maximum likelihood estimation.

For continuous outcomes, we will aim to synthesise mean differences (potentially standardised if outcome scales differ substantially) and adjust for baseline values using analysis of covariance, as recommended [31]. For binary outcomes, we will aim to synthesise relative risks or odds ratios, with the binomial nature suitably modelled using, for example, a one-step logistic regression adjusting for clustering. For any time-to-event outcome, we will aim to fit a Cox regression model (after checking for proportional hazards) in each study and then synthesise the estimated hazard ratios obtained. At the study-level, the random effects to account for heterogeneity will be assumed normally distributed allowing us to estimate the average intervention effect and its confidence interval, and the between-study variance (‘tau-squared’). To reveal the impact of heterogeneity more clearly, we will also calculate a 95% prediction interval for the intervention effect when applied in an individual clinical setting [32].

#### Examining heterogeneity and estimating intervention effects within each subgroup

To consider the causes of heterogeneity and factors that may modify the intervention effect for each outcome, for each weight management intervention we will meet the primary objectives of our project by performing the pre-specified subgroup analyses by BMI, age; ethnicity, parity, risk status of medical comorbidities in pregnancy risk; and type of intervention. To obtain the summary intervention effects in each subgroup, we will use the same random-effects meta-analysis approach as described above. Subgroup analyses, if not carefully planned, can lead to misleading results e.g. due to the play of chance with multiple testing [32]. Thus caution will be used in the interpretation of the collective set of subgroup results, and adjustment for multiple testing will be considered.

It is important to calculate the difference in intervention effect between subgroups, to ascertain if any observed differences are due to chance. This will be undertaken by extending the one-stage meta-analysis framework to include treatment-covariate interaction terms, which provide the change in intervention effect for a 1-unit change in the covariate. In doing so, we will ensure that we estimate the pooled within-trial interaction of interest separately from the across-trial (meta-regression) interaction, as recommended because the former is the desired information as it is based solely on patient-level information [33,34]. Between-study heterogeneity in the within-trial treatment-covariate will also be measured, summarised and, if necessary, accounted for in the analysis. Continuous covariates, such as BMI and age, will be analysed on their continuous scale, rather than categorisation [35]. However, to translate the results clinically, after the analysis we will report the effect of the covariate-treatment interaction on the intervention effect at clinically relevant covariate values, such normal weight values, overweight values, and obese values, and those aged under or over 18.

As a secondary analysis, we will evaluate the association between weight gain in pregnancy and adverse maternal and fetal outcomes in normal weight, overweight, and obese women. For each group separately and each outcome, we will fit a suitable regression model that accounts for clustering of patient within studies and quantifies how each 1-unit increase in weight gain changes the risk of a poor outcome. As the relationship is likely to be non-linear, we will consider non-linear trends between weight gain and outcome using fractional polynomial terms [35]. For each type of outcome, a suitable model will be used such as linear regression for continuous outcomes, or logistic regression for binary outcomes. The model will account for the clustering of patients within trials, and their treatment group allocation. Further, we will consider whether the association between weight gain and outcome interacts with whether a patient is in the intervention group or not.

#### Evaluation of potential prognostic factors for weight change in pregnancy

In secondary analyses, we will also evaluate those variables that may have a prognostic effect on gestational weight gain including age, ethnicity, underlying medical conditions like diabetes, parity, type and duration of intervention, mental health, and socioeconomic status [36]. For all candidate prognostic factors (predictors), we will perform separate analyses in each BMI cohort (normal, overweight, and obese) and analyse on the whole meta-analysis database, adjusting again for the clustering of patients within studies and accounting for heterogeneity as necessary. To obtain adjusted prognostic factor results, multivariable models will be fitted including all variables of interest, to ascertain which have independent prognostic value.

#### Network meta-analyses

We will rank the interventions according to their effectiveness using a network meta-analysis approach [37]. Under the assumption that the sets of trials in each meta-analysis are comparable, an indirect comparison will be carried out by calculating the difference in treatment effect sizes for all interventions (to get say A vs B using A vs C minus B vs C). Within-trial randomised comparisons of each study will be preserved. Our network meta-analyses will be undertaken in a frequentist framework using multivariate meta-analysis models within the STATA modules ‘network’ [38,39], which allows within-study correlations (between pairs of effect estimates from the same study) and between-study correlations to be accounted for as necessary. The consistency assumption will also be examined in this framework, for example, by comparing the difference in direct and indirect effect estimates and comparing the fit of consistency and inconsistency models. We note that where indirect comparisons have been compared to direct comparisons, over 95% concordance has been found [35,40]. Ranking will be achieved by assuming flat, uninformative prior distributions for all parameters, and thus assuming the multivariate normal sampling distribution of the pooled treatment effects is a posterior distribution with mean and variance equal to the frequentist estimates and variance-covariance matrix. Thus, approximate Bayesian inferences are then possible. One thousand draws will be made from the posterior distribution, and the treatments will be ordered according to the probability (across all draws) that they are the most effective on average.

#### Exploration of sources of bias: unavailable data and publication bias

We will explore the potential for, and possible impact of, both publication bias and unavailable data, according to recent guidelines [41]. For each analysis containing ten or more studies, the likelihood of publication bias will be investigated through the construction of contour-enhanced funnel plots and appropriate statistical tests for ‘small-study effects’; that is, the tendency for smaller studies to provide more positive findings.

In addition, for all studies where IPD were not provided to us, we will seek to extract suitable aggregate data from their study publications (such aggregate data has already been extracted from our previous HTA review). Where possible, we will then, using the two-step meta-analysis framework, combine the IPD trials with the aggregate data from other trials using suitable statistical methods to examine if conclusions change by the inclusion of additional trials [33,34]. If the inclusion of studies lacking IPD seems to have an important statistical or clinical impact, we will compare the characteristics of the studies with IPD and of those without to see if there are any key differences (such as in their quality, follow-up length, statistical methods). We recognise, however, that this approach is likely to only be achievable when examining the overall treatment effect, and our main IPD analyses of the subgroup effects are unlikely to be able to include any suitable aggregate data for subgroup effects from non-IPD studies (the very reason why we have sought IPD for meta-analysis).

### Health economic evaluation

We will develop a decision analytic simulation model as a framework for conducting cost-effectiveness and cost-utility analyses and associated value of information analyses [42,43]. The economic evaluations will inform current treatment policy in this clinical area, whilst the value of information component will serve to highlight future research needs and agendas and inform possible future research funding decisions.

The main objective of the evaluation will be to determine the characteristics of the weight management intervention(s) that are most cost-effective. Hence, the range of options (in terms of duration, frequency and intensity) for which trial data exist will be investigated.

An incremental approach will be adopted with a focus on additional costs and gain in benefits associated with a move away from current practice to alternative treatment strategies. The cost-effectiveness component of the work will report results in terms of an incremental cost-effectiveness ratio (ICER) of cost per unit of benefit gained, measured in appropriate clinical and economically relevant outcome measures.

Some limited quality of life data potentially suitable for use in a cost-utility framework are available from published sources [44,45], and so the economic evaluation will attempt additionally to present results in terms of incremental cost per quality-adjusted life year (QALY) gained.

The results will be presented using cost-effectiveness acceptability curves to reflect sampling variation and uncertainties in the appropriate threshold cost-effectiveness value. We shall also include a value of information analysis to quantify the total uncertainty in terms of the value of removing that uncertainty. In addition to this probabilistic sensitivity analysis on our base-case model, we shall include a range of alternative analyses to explore the robustness of these results to plausible variations in key assumptions and variations in the analytical methods used and to consider the broader issue of the generalisability of the results.

## Discussion

The IPD meta-analysis will allow us to identify and subsequently target the interventions to those groups that show clear benefit with weight management in pregnancy. It has greater power to detect any differential treatment effect across groups as it can model individual risk status (prognostic factor values) across participants within trials and thus explain variability in outcomes at the patient-level [16]. In contrast, aggregate data meta-analysis can only model average risk status values across studies and thus only explain variation in summary outcomes at the study-level. Availability of IPD alleviates the need to use published results and is thus less likely to be affected by selective and biased reporting than an aggregate data meta-analysis. It also has the potential to assess longer follow-up and include more participants and more outcomes than reported in the original publication.

Weight gain in pregnancy varies with age, ethnicity, and parity. Pregnancy during adolescence may alter normal growth processes and increase the risk of the mothers becoming overweight or obese [46]. Adolescent mothers also retain more weight postpartum than mature control subjects [46]. Therefore, inclusion of a large number of pregnant adolescents may overestimate postpartum weight changes or the risk of becoming overweight and thus bias estimates for mature women. In the US, immigrant women are known to have less gestational weight gain but the same rate of complications in pregnancy compared to the domestic population [47]. Ethnic differences in the relationship between weight gain and complications need further investigation.

The trials identified in our previous HTA systematic review on diet and lifestyle interventions in pregnancy were powered to detect an overall treatment effect, but not to detect a subgroup effect. The costs and time to undertake a new trial for this purpose would be immense.

One of the key recommendations that arose from the study level meta-analysis of dietary and physical activity interventions in pregnancy was the need to synthesise patient level data to assess any differential effect of the benefits observed with interventions in various subgroups [21,27]. Such questions are difficult to answer using extracted results from trial publications, as patient-level information is no longer available and subgroup effects (‘treatment-covariate interactions’) are rarely reported in sufficient detail. Furthermore, meta-regression examining the across-trial association between overall treatment effect and average patient characteristics (e.g. mean age) generally has low power to detect genuine subgroup effects and is also prone to study-level confounding [33,48].

We have chosen composite maternal and fetal outcomes to assess the effects of interventions in pregnancy as it is difficult to identify one clinically important outcome. The components of the composite maternal and fetal outcomes were identified by Delphic survey of experts ensuring face validity of the components. Through our systematic review of randomised trials, we have shown that there is an association between change in the individual outcomes in the same direction and weight management interventions, thereby ensuring content validity of the chosen composite outcome measure [27].

Our collaborative group has provisional support so far to include over 9,000 women. In contrast, single trials in this field have so far included much smaller number of women, with a median number of 137 women (smallest *n* =12; largest *n* =1,500). Thus, there is over a 50-fold increase in the sample size for our IPD project compared to the median number in the trials. We recognise that there is additional variability in an IPD meta-analysis due to clustering of patients within studies and heterogeneity in effects across studies. However, compared to a single trial that typically has 137 women, the provision of 9,000 patients within our IPD meta-analysis will dramatically improve upon single-trial research.

## Abbreviations

BMI: body mass index; CDSR: Cochrane Database of Systematic Reviews; CENTRAL: Cochrane Central Register of Controlled Trials; DARE: Database of Abstracts of Reviews of Effects; HTA: Health Technology Assessment Database; ICER: incremental cost-effectiveness ratio; IOM: Institute of Medicine; IPD: individual patient data; i-WIP: International Weight Management in Pregnancy; MID: minimally important difference; NICE: National Institute for Health and Care Excellence; NHS: National Health Service; PHIAC: Public Health Interventions Advisory Committee; QALY: quality-adjusted life year; SD: standard deviation; SIGLE: Systems for Information in Grey Literature; UK: United Kingdom; US: United States; VOI: value of information; WHO: World Health Organization.

## Competing interests

The authors declare that they have no competing interests.

## Authors’ contributions

AR was involved in the concept and the design of the study and planned and wrote the initial protocol. She also participated in face-to-face meetings and/or teleconferences to discuss protocol, design of the meta-analysis, the choice of outcome measures and analysis strategies. ST was involved in the concept and the design of the study, and planned and wrote the initial protocol. She also participated in face-to-face meetings and/or teleconferences to discuss protocol, design of the meta-analysis, the choice of outcome measures and analysis strategies. RR was involved in the concept and the design of the study, and wrote a significantly part of the protocol. He also participated in face-to-face meetings and/or teleconferences to discuss the protocol, design of the meta-analysis, the choice of outcome measures and analysis strategies. KK was involved in the concept and the design of the study, and contributed significantly to the planning and writing of the protocol. He also participated in face-to-face meetings and/or teleconferences to discuss the protocol, design of the meta-analysis, the choice of outcome measures and analysis strategies. BWM was involved in the concept and the design of the study, and contributed significantly to the planning and writing of the protocol. He also participated in face-to-face meetings and/or teleconferences to discuss the protocol, design of the meta-analysis, the choice of outcome measures and analysis strategies. ER was involved in the concept and the design of the study, and contributed significantly to the planning and writing of the protocol. She also participated in face-to-face meetings and/or teleconferences to discuss the protocol, design of the meta-analysis, the choice of outcome measures and analysis strategies. MvP contributed significantly to the planning and writing of the protocol. She also participated in face-to-face meetings to discuss the protocol, design of the meta-analysis, the choice of outcome measures and analysis strategies. GR contributed significantly to the planning and writing of the protocol. He also participated in face-to-face meetings and/or teleconferences to discuss the protocol, design of the meta-analysis, the choice of outcome measures and analysis strategies. SK contributed significantly to the planning and writing of the protocol. She also participated in face-to-face meetings and/or teleconferences to discuss the protocol, design of the meta-analysis, the choice of outcome measures and analysis strategies. CdG was involved in the concept and the design of the study, and contributed significantly to the planning and writing of the protocol. SY contributed significantly to the planning and writing of the protocol. EM contributed significantly to the planning and writing of the protocol. She also participated in face-to-face meetings and/or teleconferences to discuss the protocol, design of the meta-analysis, the choice of outcome measures and analysis strategies. FM contributed significantly to the planning and writing of the protocol. She also participated in face-to-face meetings and/or teleconferences to discuss the protocol, design of the meta-analysis, the choice of outcome measures and analysis strategies. LP was contributed significantly to the planning and writing of the protocol. She also participated in face-to-face meetings and/or teleconferences to discuss the protocol, design of the meta-analysis, the choice of outcome measures and analysis strategies. TR contributed significantly to the planning and writing of the protocol. She also participated in face-to-face meetings and/or teleconferences to discuss the protocol, design of the meta-analysis, the choice of outcome measures and analysis strategies. AC contributed significantly to the planning and writing of the protocol. He also participated in face-to-face meetings and/or teleconferences to discuss the protocol, design of the meta-analysis, the choice of outcome measures and analysis strategies. All authors critically reviewed the subsequent versions of the manuscript and approved the final manuscript.

## Figures and Tables

**Table 5:** i-WIP collaborators

**Trial**	**Year**	**Corresponding author**	**Country**	**Affiliation**
Althuizen	2012	Mireille van Poppel	Amsterdam, Netherlands	Department of Public and Occupational Health, EMGO + Institute for Health and Care Research, VU University Medical Center, Amsterdam
Barakat	2009, 2011, 2013	Ruben Barakat Carballo	Madrid, Spain	Facultad de Ciencias de la Actividad Fı’sica y del Deporte-INEF, Universidad Polite’cnica de Madrid
Bogaerts	2012	Annick Bogaerts	Leuven, Belgium	Division of Mother and Child, Department of Obstetrics and Gynaecology, Limburg Catholic University College Hasselt and University Hospitals KU Leuven
Cavalcante	2009	Jose G Cecatti	Sao Paulo, Brazil	Department of Obstetrics and Gynecology, School of Medical Sciences, University of Campinas (UNICAMP)
Clapp	1997, 2000	Beth Lopez	Cleveland, USA	Departments of Reproductive Biology and Obstetrics and Gynecology and the Schwartz Center for Metabolism and Nutrition, Case Western Reserve University and MetroHealth Medical Center
Dodd	2014	Jodie Dodd	Adelaide, Australia	Discipline of Obstetrics and Gynaecology, School of Paediatrics and Reproductive Health, The University of Adelaide
El Beltagy	2013	Nermeen El Beltagy	Alexandria, Egypt	Department of Obstetrics and Gynecology, Alexandria University
Guelinckx	2010	Roland Devlieger	Leuven, Belgium	Division of Mother and Child, Department of Obstetrics and Gynaecology, University Hospitals KU Leuven
Haakstad	2011	Lene Haakstad	Oslo, Norway	Norwegian School of Sport Sciences, Department of Sports Medicine
Hui	2006, 2011	Gary Shen	Manitoba, Canada	Department of Internal Medicine, University of Manitoba, Winnipeg
Jeffries	2009	Alexis Shub	Melbourne, Australia	Department of Obstetrics and Gynaecology, University of Melbourne
Khaledan	2010	Narges Motahari	Babolsar, Iran	Dept. Physiology, School of Physical Education, Mazandaran University
Khoury	2005	Janette Khoury	Oslo, Norway	Department of Obstetrics and Gynecology, National University Hospital
Luoto	2011	Riitta Luoto	Helsinki, Finland	UKK Institute for Health Promotion Research
Nashimento	2011	Jose G Cecatti, PhD MD	Sao Paulo, Brazil	Department of Obstetrics and Gynecology, School of Medical Sciences, University of Campinas (UNICAMP)
Ong	2009	Kym Guelfi	Crawley, Australia	School of Sport Science, Exercise and Health, The University of Western Australia
Oostdam	2009	Mireille van Poppel	Amsterdam, Netherlands	Department of Public and Occupational Health, EMGO + Institute for Health and Care Research, VU University Medical Center, Amsterdam
Petrella	2013	Fabio Facchinetti	Modena, Italy	Mother-Infant Department, University of Modena and Reggio Emilia
Phelan	2011	Suzanne Phelan	San Luis Obispo, USA	Kinesiology Department, California Polytechnic State University
Poston	2013	Lucilla Poston	London, UK	King’s College London, Division of Women’s Health, Women’s Health Academic Centre
Prevedel	2003	Tânia T Scudeller Prevedel	Sao Paulo, Brazil	Department of Obstetrics, Faculty of Medicine, Botucatu
Rauh	2013	Kathrin Rauh	Munich, Germany	Else Kroener-Fresenius-Center for Nutritional Medicine, Chair of Nutritional Medicine, Technische Universität München
Renault	2013	Kristina Renault	Copenhagen, Denmark	Departments of Obstetrics and Gynecology, Hvidovre Hospital, University of Copenhagen
Sagedal	2014	Linda Reme Sagedal	Kristiansand, Norway	Department of Obstetrics and Gynecology, Sorlandet Hospital
Stafne	2012	Signe Nilssen Stafne and Siv Mørkved	Trondheim, Norway	Department of Public Health and General Practice, Faculty of Medicine, Norwegian University of Science and Technology
Vesco	2013	Kimberly Vesco	Portland, USA	Center for Health Research, Portland
Vinter	2011	Christina Vinter	Odense, Denmark	Department of Gynecology and Obstetrics, Odense University Hospital
Vitolo	2011	Vitolo	Porto Alegre, Brazil	Department of Nutrition and the Graduate Program in Health Sciences, Federal University of Health Sciences of Porto Alegre
Walsh	2012	Fionnuala M McAuliffe	Dublin, Ireland	UCD Obstetrics and Gynaecology, School of Medicine and Medical Science, University College Dublin, National Maternity Hospital, Dublin, Ireland
Wolff	2008	Arne Astrup	Copenhagen, Denmark	Department of Human Nutrition, Faculty of Life Science, Copenhagen University
Yeo	2000 and 2012	Seonae Yeo	Chapel Hill, North Carolina, USA	School of Nursing, University of North Carolina at Chapel Hill
