# Erratum to: Study protocol: differential effects of diet and physical activity based interventions in pregnancy on maternal and fetal outcomes: individual patient data (IPD) meta-analysis and health economic evaluation

**DOI:** 10.1186/s13643-015-0079-2

**Published:** 2015-07-30

**Authors:** Anneloes E. Ruifrok, Ewelina Rogozinska, Mireille N. M. van Poppel, Girish Rayanagoudar, Sally Kerry, Christianne J. M. de Groot, SeonAe Yeo, Emma Molyneaux, Ruben Barakat Carballo, Maria Perales, Annick Bogaerts, Jose G. Cecatti, Fernanda Surita, Jodie Dodd, Julie Owens, Nermeen El Beltagy, Roland Devlieger, Helena Teede, Cheryce Harrison, Lene Haakstad, Garry X. Shen, Alexis Shub, Narges Motahari, Janette Khoury, Serena Tonstad, Riitta Luoto, Tarja I. Kinnunen, Kym Guelfi, Fabio Facchinetti, Elisabetta Petrella, Suzanne Phelan, Tânia T. Scudeller, Kathrin Rauh, Hans Hauner, Kristina Renault, Linda Reme Sagedal, Ingvild Vistad, Signe Nilssen Stafne, Siv Mørkved, Kjell Åsmund Salvesen, Christina Vinter, Marcia Vitolo, Arne Astrup, Nina Rica Wium Geiker, Fionnuala McAuliffe, Lucilla Poston, Tracy Roberts, Richard D. Riley, Arri Coomarasamy, Khalid S. Khan, Ben Willem Mol, Shakila Thangaratinam

**Affiliations:** Department of Obstetrics and Gynecology, Academic Medical Centre, Amsterdam, The Netherlands; Department of Obstetrics and Gynaecology, Faculty of Medicine, VU University Medical Center, Amsterdam, The Netherlands; Women’s Health Research Unit, Barts and The London School of Medicine and Dentistry, Queen Mary University of London, London, UK; Department of Public and Occupational Health, EMGO+ Institute for Health and Care Research, VU University Medical Center, Amsterdam, The Netherlands; Multidisciplinary Evidence Synthesis Hub (mEsh), Barts and The London School of Medicine and Dentistry, Queen Mary University of London, London, UK; University of North Carolina at Chapel Hill, School of Nursing, Chapel Hill, NC USA; Section of Women’s Mental Health, Health Service and Population Research Department, Institute of Psychiatry, King’s College London, London, UK; School of Medicine & Medical Science, UCD Institute of Food and Health, Dublin, Ireland; Division of Women’s Health, Women’s Health Academic Centre, King’s College London, St. Thomas’ Hospital, London, UK; Health Economics Unit, School of Health and Population Sciences, College of Medical and Dental Sciences, University of Birmingham, Birmingham, UK; School of Health and Population Sciences, College of Medical and Dental Sciences, University of Birmingham, Birmingham, UK; School of Clinical and Experimental Medicine, College of Medical and Dental Sciences, University of Birmingham, Birmingham, UK; Robinson Institute, School of Paediatrics and Reproductive Health, University of Adelaide, Adelaide, Australia; Facultad de Ciencias de la Actividad Fısica y del Deporte-INEF, Universidad Politecnica de Madrid, Madrid, Spain; Division of Mother and Child, Department of Obstetrics and Gynaecology, University Colleges Leuven-Limburg, Hasselt and University Hospitals KU Leuven, Leuven, Belgium; Department of Obstetrics and Gynecology, School of Medical Sciences, University of Campinas (UNICAMP), Campinas, Brazil; Discipline of Obstetrics and Gynaecology, School of Paediatrics and Reproductive Health, The University of Adelaide, Adelaide, Australia; Department of Obstetrics and Gynecology, Alexandria University, Alexandria, Egypt; Monash Centre for Health Research and Implementation -MCHRI, School of Public Health Monash University, Melbourne, Australia; Norwegian School of Sport Sciences, Department of Sports Medicine, Oslo, Norway; Department of Internal Medicine, University of Manitoba, Winnipeg, Canada; Department of Obstetrics and Gynaecology, University of Melbourne, Melbourne, Australia; Department of Sport Physiology, Faculty of Physical Education and Sport Sciences, Mazandaran University, Babolsar, Iran; Department of Obstetrics and Gynecology, Oslo University Hospital, Oslo, Norway; UKK Institute for Health Promotion Research, Tampere, Finland; School of Health Sciences, University of Tampere, Tampere, Finland; School of Sport Science, Exercise and Health, The University of Western Australia, Perth, Australia; Mother-Infant Department, University of Modena and Reggio Emilia, Modena, Italy; Kinesiology Department, California Polytechnic State University, San Luis Obispo, USA; Department of Health Sciences, Physical Therapy Course, São Paulo Federal University/Unifesp, Santos, Brazil; Else Kroener-Fresenius-Center for Nutritional Medicine, Chair of Nutritional Medicine, Technische Universität München, Munich, Germany; Competence Centre for Nutrition (KErn), Freising, Germany; Departments of Obstetrics and Gynecology, Hvidovre Hospital, University of Copenhagen, Copenhagen, Denmark; Department of Obstetrics and Gynecology, Odense University Hospital, University of Southern Denmark, Odense, Denmark; Department of Obstetrics and Gynecology, Sorlandet Hospital, Kristiansand, Norway; Department of Public Health and General Practice, Faculty of Medicine, Norwegian University of Science and Technology, Trondheim, Norway; Clinical Services, St. Olavs Hospital, Trondheim University Hospital, Trondheim, Norway; Department of Obstetrics and Gynaecology, Clinical Sciences, Lund University, Lund, Sweden; Department of Laboratory Medicine Children’s and Women’s Health, Faculty of Medicine, Norwegian University of Science and Technology, Trondheim, Norway; Department of Nutrition and the Graduate Program in Health Sciences, Federal University of Health Sciences of Porto Alegre, Porto Alegre, Brazil; Department of Human Nutrition, Faculty of Life Science, Copenhagen University Copenhagen, Copenhagen, Denmark; Nutritional Research Unit, Copenhagen University Hospital Herlev, Herlev, Denmark

## Erratum

After publication of this work [1], we noted that we inadvertently failed to include the complete list of all coauthors and that sample sizes of some of the trials listed in Table two were incorrect.

The full list of authors has now been added and includes the names of all authors within the i-WIP Collaborative Network. The Authors’ contributions and competing interests section modified accordingly. We are publishing this erratum to update the author list, which is as follows:

Anneloes E Ruifrok, Ewelina Rogozinska, Mireille NM van Poppel, Girish Rayanagoudar, Sally Kerry, Christianne JM de Groot, SeonAe Yeo, Emma Molyneaux, Fionnuala McAuliffe, Lucilla Poston, Tracy Roberts, Richard D Riley, Arri Coomarasamy, Khalid Khan, Ben Willem Mol, Ruben Barakat Carballo, Maria Perales, Annick Bogaerts, Jose G Cecatti, Fernanda Surita, Jodie Dodd, Julie Owens, Nermeen El Beltagy, Roland Devlieger, Helena Teede, Cheryce Harrison, Lene Haakstad, Garry X Shen, Alexis Shub, Narges Motahari, Janette Khoury, Serena Tonstad, Riitta Luoto, Tarja I Kinnunen, Kym Guelfi, Fabio Facchinetti, Elisabetta Petrella, Suzanne Phelan, Tânia T Scudeller, Kathrin Rauh, Hans Hauner, Kristina Renault, Linda Reme Sagedal, Ingvild Vistad, Signe Nilssen Stafne, Siv Mørkved, Kjell Åsmund Salvesen, Christina Vinter, Marcia Vitolo, Arne Astrup, Nina Rica Wium Geiker and Shakila Thangaratinam.

The sample sizes of trials included in Table two have been corrected (Table 1). We are publishing this erratum to update these trial sample sizes, which include Dodd 2014 (*n* = 2212), Prevedel 2003 (*n* = 41), Renault 2013 (*n* = 425), Stafne 2012 (*n* = 855), Vinter 2011 (*n* = 360), Walsh 2012 (*n* = 800) and Wolff 2008 (*n* = 66).

**Table 1:** Studies with provisional support and consideration to share individual patient data

Study Year	Country	Study Characteristics	Outcomes		Sample size
			Maternal	Fetal	
Althuizen 2012	Netherlands	Ethnically diverse , no BMI restrictions, age n.r., GA at inclusion < 14 wks, glucose status n.r., other risk factors: n.r.	GWG, GDM, preterm delivery, CS	birth weight, macrosomia	269
Barakat 2009	Spain	Caucasian, BMI restrictions n.r., age 25–35 yrs, GA at inclusion n.r. (total at least 26 wks intervention), glucose status n.r., no known pre-existing health problems	GWG, GA, preterm delivery	birth weight, LGA, SGA, AS, macrosomia (>4000g)	142
Barakat 2011	Spain	Spanish (white), BMI restrictions n.r., age 23–38 yrs, GA at inclusion 1st prenatal visit, glucose status n.r., no known pre-existing health problems	GWG, GA CS, vaginal delivery	birth weight, AS	80
Barakat 2013	Spain	Caucasian, no BMI restrictions, age n.r., GA at inclusion <10 wks, glucose status n.r., no known pre-existing health problems	GWG, GA, GDM, PIH, preterm delivery	birth weight, AS	765
Bogaerts 2012	Belgium	Ethnically diverse , BMI ≥ 29 kg/m2, age n.r., GA at inclusion < 15 wks, nondiabetic, other risk factors: n.r	GWG, GA, PE, PIH, GDM, IOL, CS, vaginal delivery	birth weight, AS	197
Cavalcante 2009	Brazil	Race n.r., no morbid obesity, age restrictions n.r., GA at inclusion 16–20 wks, glucose status n.r., no known pre-existing health problems	GWG, preterm delivery	birth weight	71
Clapp 1997	USA	Race n.r., no morbid obesity, age restrictions n.r., GA at inclusion 8 wks, glucose status n.r., no known pre-existing health problems	GWG	birth weight	51
Clapp 2000	USA	Race n.r., no morbid obesity, age restrictions n.r., GA at inclusion 8 wks, glucose status n.r., no known pre-existing health problems	GWG, GA	birth weight	12
Dodd 2014	Australia	Race n.r., BMI ≥ 25 kg/m^2^, age restrictions n.r., GA at inclusion <20 wks, nondiabetic, other risk factors: n.r.	PE, PIH, GDM, IOL, CS, Preterm delivery	LGA, macrosomia (>4000g), hypoglycaemia, shoulder dystocia, admission to NICU	2,212
El Beltagy 2013	Egypt	Race n.r., BMI: obese, age restrictions n.r., GA at inclusion: first antenatal visit, glucose status n.r., other risk factors: n.r.	GWG, GDM	birth weight, macrosomia	100
Grant 2013	Canada	Race : predominantly non-Caucasian, BMI restrictions n.r., age >18 yrs, GA at inclusion n.r., glucose status: impaired glucose tolerance or GDM, no known pre-existing health problems	GWG	birth weight, macrosomia	47
Guelinckx 2010	Belgium	Caucasian, BMI ≥ 29 kg/m^2^, age restrictions n.r., GA at inclusion <15 wks, nondiabetic, no known pre-existing health problems	GWG, GA, PE, PIH, IOL, CS	birth weight, LGA	85
Haakstad 2011	Norway	Race n.r., BMI restrictions n.r., age restrictions n.r., GA at inclusion <24 wks, glucose status n.r., no known pre-existing health problems	GWG		105
Hui 2006	Canada	Ethnically diverse, BMI restrictions n.r., age restrictions n.r., GA at inclusion <26 wks, nondiabetic, no known pre-existing health problems	GWG, GA, GDM	birth weight, LGA	45
Hui 2011	Canada	Race n.r., BMI restrictions n.r., age restrictions n.r., GA at inclusion 20–26 wks, nondiabetic, no known pre-existing health problems	GWG, GA, GDM, CS	birth weight, LGA	224
Jackson 2010	USA	Ethnically diverse, BMI restrictions n.r., age >18 yrs, GA at inclusion <26 wks, glucose status n.r., other risk factors: n.r.	GWG		321
Jeffries 2009	Australia	Race n.r., BMI restrictions none, age >18 - <45 yrs, GA at inclusion <14 wks, nondiabetic, other risk factors: n.r.	GWG, PE, PIH, GDM , preterm delivery, CS	birth weight, LGA, SGA, hypoglycaemia, shoulder dystocia	236
Khaledan 2010	Iran	Race n.r., BMI restrictions n.r., age restrictions n.r., GA at inclusion 24–32 wks, no Diabetes Mellitus type 1 (DM1) with poor control, no known pre-existing health problems	GWG, GA, CS	birth weight	39
Khoury 2005	Norway	Caucasian, BMI 19–32 kg/m^2^. age 21–38 yrs, GA at inclusion 17–20 wks, nondiabetic, no known pre-existing health problems	GWG, PE, preterm delivery	birth weight, SGA, intra-uterine death	290
Luoto 2011	Finland	Race n.r., BMI >17 kg/m^2^, age >18 yrs, GA at inclusion 8–12 wks, nondiabetic, no known pre-existing health problems	GWG, GA, PE, GDM	birth weight, LGA, SGA	399
Nascimento 2011	Brazil	Race n.r., BMI >26 kg/m^2^, age >18 yrs, GA at inclusion 14–24 wks, nondiabetic, no known pre-existing health problems	GWG, PIH, GDM, CS	birth weight, AS, LGA, SGA	82
Ong 2009	Australia	Race n.r., obese, age restrictions n.r., GA at inclusion 18 wks, nondiabetic, other risk factors: n.r.	GWG		12
Oostdam 2012	Netherlands	Ethnically diverse, BMI ≥ 25.0 kg/m^2^, age > 18 yrs, GA at inclusion <20 wks, nondiabetic, no known pre-existing health problems	GWG, GDM	birth weight	124
Petrella 2013	Italy	Ethnically diverse , BMI ≥ 25.0 kg/m^2^, age > 18 yrs, GA at inclusion 12 wks, nondiabetic, no known pre-existing health problems	GWG, GDM, PIH, preterm delivery		63
Phelan 2011	USA	Ethnically diverse, BMI ≥19.8-26.0 kg/m^2^, age >18 yrs, GA at inclusion 10–16 wks, glucose status n.r., no known pre-existing health problems	GWG, GA, PE, PIH, GDM, preterm delivery, CS	birth weight, macrosomia, birth weight <2500g	401
Poston 2013	United Kingdom	Race: n.r., BMI ≥30 kg/m^2^, age restrictions n.r., GA at inclusion >15^+0^ weeks and <17^+6^, , nondiabetic, no known pre-existing health problems	GA, GWG, PE, GDM, mode of delivery	Birth weight, macrosomia, still birth	183
Prevedel 2003	Brazil	Race: n.r., BMI restrictions n.r., age restrictions n.r. (primiparous or adolescents), GA at inclusion 16–20 wks, glucose status n.a., no known pre-existing health problems	GWG, preterm delivery	birth weight, SGA	41
Rauh 2013	Germany	Race: n.r., BMI ≥18.5 kg/m^2^, age ≥18 yrs, GA at inclusion <18 wks, nondiabetic, no known pre-existing health problems	GWG, GDM, IOL, CS, preterm delivery	Birth weight LGA, SGA	250
Renault 2013	Denmark	Race: predominantly Caucasian, BMI ≥30 kg/m^2^, age >18 yrs, GA at inclusion <16 wks, nondiabetic, no known pre-existing health problems	GWG, GDM, PIH,PE,IOL, CS, preterm delivery	Birth weight, SGA, LGA, Birth weight >4000g	425
Sagedal 2014	Norway	Race: n.r., BMI ≥19 kg/m^2^, age ≥18 yrs, GA at inclusion <20 wks, nondiabetic, no known pre-existing health problems	GWG, GDM, CS	LGA	606
Stafne 2012	Norway	White, no BMI restrictions, age >18 yrs, GA at inclusion 18–22 wks, nondiabetic, no known pre-existing health problems	GA, PE, PIH, GDM, CS	birth weight, AS, LGA, admission to NICU	855
Vesco 2013	USA	Race: n.r., BMI ≥30 kg/m^2^, age n.r., GA at inclusion <20 wks, nondiabetic, no known pre-existing health problems	GWG, GA, PE, PIH, GDM, CS, preterm delivery	birth weight, LGA, SGA, macrosomia (4000g)	114
Vinter 2011	Denmark	Caucasian, BMI 30–45 kg/m^2^, age 18–40 yrs, GA at inclusion 10–14 wks, nondiabetic, no known pre-existing health problems	GWG, PE, PIH, GDM, CS	LGA, admission to NICU	360
Vitolo 2011	Brasil	Race: n.r., BMI restrictions: none, age <35yrs, GA at inclusion 10–29 wks, nondiagetic, no known pre-existing health problems	GWG,PE, PIH, GDM, preterm birth	birth weight	315
Walsh 2012	Ireland	Race: n.r., BMI restrictions n.r., age >18 yrs, GA at inclusion < 18 wks, nondiabetic, no known pre-existing health problems	GWG, GA, preterm delivery, IOL, CS	birth weight, macrosomia	800
Wolff 2008	Denmark	Caucasian, BMI ≥30 kg/m^2^, age >18 - <45 yrs, GA at inclusion <18 wks, nondiabetic, no known pre-existing health problems	GWG PE, PIH, GDM , CS	birth weight	66
Yeo 2012	USA	Ethnically diverse, BMI >19.8 kg/m^2^, no age restrictions, GA at inclusion 18 wks, nondiabetic, no known pre-existing health problems	GWG, PE, PIH	birth weight	17

*AS* Apgar score, *CS* Caesarean section, *GA* Gestational Age, *GDM* Gestational diabetes mellitus, *GWG* Gestational weight gain, *IOL* Induction of labour, *LGA* Large for gestational age, *NICU* Neonatal Intensive Care Unit, *n.r.* not reported, *PE* Pre eclampsia, *PIH* Pregnancy Induced hypertension, *RDS* Respiratory Distress Syndrome, *SGA* Small for gestational age
